# Circulating messenger RNA variants as a potential biomarker for surveillance of hepatocellular carcinoma

**DOI:** 10.3389/fonc.2022.963641

**Published:** 2022-12-13

**Authors:** Timothy Block, Daniel Zezulinski, David E. Kaplan, Jingqiao Lu, Samantha Zanine, Tingting Zhan, Cataldo Doria, Aejaz Sayeed

**Affiliations:** ^1^ Department of Translational Medicine, Baruch S. Blumberg Institute, Doylestown, PA, United States; ^2^ Division of Gastroenterology and Hepatology, University of Pennsylvania Perelman School of Medicine and The Corporal Michael J. Crescenz Veterans Administration Hospital, Philadelphia, PA, United States; ^3^ Ray Biotech Life Inc., Peachtree Corners, GA, United States; ^4^ Department of Mechanical Engineering, Pennsylvania State University, PA, United States; ^5^ Division of Biostatistics, Department of Pharmacology and Experimental Therapeutics, Thomas Jefferson University, Philadelphia PA, United States; ^6^ CHS Liver and Pancreas Centers of Excellence, Capital Health Cancer Center, One Capital Way, Pennington, NJ, United States

**Keywords:** circulating mRNA variants, hepatocellular carcinoma, liver cirrhosis, extracellular vesicles, HCC surveillance and early detection

## Abstract

**Background and rationale:**

Liver derived messenger ribonucleic acid (mRNA) transcripts were reported to be elevated in the circulation of hepatocellular carcinoma (HCC) patients. We now report the detection of high-risk mRNA variants exclusively in the circulation of HCC patients. Numerous genomic alleles such as single nucleotide polymorphisms (SNPs), nucleotide insertions and deletions (called Indels), splicing variants in many genes, have been associated with elevated risk of cancer. Our findings potentially offer a novel non-invasive platform for HCC surveillance and early detection.

**Approach:**

RNAseq analysis was carried out in the plasma of 14 individuals with a diagnosis of HCC, 8 with LC and no HCC, and 6 with no liver disease diagnosis. RNA from 6 matching tumors and 5 circulating extracellular vesicle (EV) samples from 14 of those with HCC was also analyzed. Specimens from two cholangiocarcinoma (CCA) patients were also included in our study. HCC specific SNPs and Indels referred as “variants” were identified using GATK HaplotypeCaller and annotated by SnpEff to filter out high risk variants.

**Results:**

The variant calling on all RNA samples enabled the detection of 5.2 million SNPs, 0.91 million insertions and 0.81 million deletions. RNAseq analyses in tumors, normal liver tissue, plasma, and plasma derived EVs led to the detection of 5480 high-risk tumor specific mRNA variants in the circulation of HCC patients. These variants are concurrently detected in tumors and plasma samples or tumors and EVs from HCC patients, but none of these were detected in normal liver, plasma of LC patients or normal healthy individuals. Our results demonstrate selective detection of concordant high-risk HCC-specific mRNA variants in free plasma, plasma derived EVs and tumors of HCC patients. The variants comprise of splicing, frameshift, fusion and single nucleotide alterations and correspond to cancer and tumor metabolism pathways. Detection of these high-risk variants in matching specimens from same subjects with an enrichment in circulating EVs is remarkable. Validation of these HCC selective ctmRNA variants in larger patient cohorts is likely to identify a predictive set of ctmRNA with high diagnostic performance and thus offer a novel non-invasive serology-based biomarker for HCC.

## Introduction

Hepatocellular carcinoma (HCC) represents the fourth most deadly cancer worldwide and projected to become the 3rd leading cause of cancer related deaths by 2030 (1–3). Rising HCC incidence has been attributed to increasing prevalence of chronic liver disease (CLD) specifically non- alcoholic steatohepatitis (NASH) and viral hepatitis (4–6), which can progress to liver cirrhosis (LC) detected in up to 90% of HCC patients (7). Development of cirrhosis significantly increases risk of HCC (8). Though traditionally HCC is known to arise in the context of hepatitis B (HBV) and hepatitis C (HCV) viral infections, metabolic associated fatty liver disease (MAFLD) and NASH are becoming more prominent risk factors for HCC (9–11). Because early-stage tumor diagnosis improves options for potentially curative therapy and thereby improves overall survival (12), AASLD guidelines recommend surveillance in CLD patients at risk for HCC (8, 13) with liver ultrasound (US) and serum alpha-fetoprotein (AFP) test conducted every 6 months (14). Ultrasound plus AFP remains relatively insensitive for early detection identifying only 63% of early-stage cancers (15). Alternative surveillance biomarkers, Lens-culinaris agglutinin-reactive AFP, known more commonly as AFP-L3, and des-carboxy prothrombin (DCP), used in Japan for screening, are more specific but less sensitive biomarkers and therefore less utilized (16). Recently, GALAD score (Gender, Age, AFP-L3, AFP, DCP) has been proposed which combines simple demographic data with serum biomarkers and predicts the future probability of developing HCC in CLD patients (17, 18). Despite these advances, surveillance is suboptimal not only due to low sensitivity of existing methods of early detection but also due to barriers in access to quality imaging and biomarkers (13, 19, 20). When ultrasound or AFP detect abnormalities, AASLD and EASL guidelines recommend magnetic resonance imaging (MRI) using extracellular or hepatobiliary contrast media or dynamic computed tomography as diagnostic testing using the Liver Imaging Reporting and Data Systems (LI-RADS) grading system (21). However, vascular imaging provides little information about tumor biology, patient prognosis or likelihood of response to therapy. Since a diagnosis of HCC is most often made radiologically without a biopsy, key tumor molecular information rarely is available to optimize therapy. The limitations of testing lead to a critical need for improving HCC surveillance and early detection (15, 22, 23).

Cell-free DNA (cfDNA) in the blood provides a useful non-invasive diagnostic analyte for cancer although for early detection, both technical and biological factors introduce challenges to the detection of mutant DNA in plasma and its interpretation (24–26). Detection of tumors is also possible through analysis of circulating tumor cells (CTCs). However, density of CTCs in circulation has been observed to be very low presumably because only large tumors release CTCs (27). cfDNA analysis in cancer clinical trials has suggested promising results in early detection, real time monitoring and management (28). Fragmentation patterns of cfDNA combined with mutations in cfDNA has been reported to increase the sensitivity of cancer detection (29). However, the mechanisms of release and degradation of cfDNA, and the factors that affect the representation of circulating tumor DNA (ctDNA) in plasma, are poorly understood. Additionally there are challenges in differentiating between potential tumors within a mixed liquid biopsy due to the low allelic frequency have been reported (25, 26). Circulating mRNA, without the copy number limitations of DNA, provides an alternative platform for use in cancer early detection and diagnostics.

Cancer is a multistep process driven by cumulative acquisition of both germline (inherited) and somatic (not inherited) mutations leading to the transformation of normal hepatocytes into malignant clones. Genetic variants can be classified into several categories, including single nucleotide polymorphisms (SNPs), small insertions and deletions (Indels), and structural variants (30). SNPs account for >90% of allelic disparities scattered throughout the human genome and nonsynonymous SNPs alter the amino acid sequence as well as potentially affect protein structure and function (31, 32). We have reported the detection of tumor specific mRNA transcripts in the circulation of HCC patients by RNAseq (33). Here we report the detection of high-risk variants comprising of both SNPs and indels in the circulating mRNA of cancer patients using RNAseq. Mutation profiling by NGS platforms can potentially lead to false-positive results due to errors introduced during library preparation and subsequent sequencing steps. These challenges have necessitated the use of multiple mutation-enrichment methods like GATK (34) and SNPeff tools (35, 36). It has been suggested that comparison of paired tumor and plasma samples represents an important prerequisite to evaluate the diagnostic accuracy of analytical platforms, especially for variants with low allele frequency (37, 38). Profiling the mutational landscape reflected in mRNA of the tumor in parallel with that of the circulation from same patient may identify critical concordant RNA variants which could potentially be used to develop a liquid biopsy platform for surveillance and early HCC detection. Here we demonstrate the identification of high-risk HCC-specific mRNA variants present exclusively in tumors and plasma of HCC patients, but not detectable in LC patients without cancer or normal healthy individuals. These circulating tumor-specific mRNA variants are derived from RNAseq analysis of total RNA using GATK and SnpEff tools, further validation and characterization of these RNA variants is essential. Our study potentially offers the promise of a novel analyte to be used in non-invasive detection of high-risk cancer specific mRNA variants and may facilitate effective surveillance and screening of ever-increasing numbers of patients with chronic liver disease.

## Materials and methods

### Human subjects

Samples were acquired from commercial vendor, Biochemed Inc and from our collaborators at The University of Pennsylvania (UPENN) and Capital Health Cancer research Institute. All samples were acquired before any therapeutic regimen was initiated. Patient plasma samples were collected at the time of diagnosis and acquired from treatment naïve adult consenting donors diagnosed with either hepatocellular carcinoma (HCC), cholangiocarcinoma or liver cirrhosis. Tumor tissues were harvested *via* biopsy or surgical resection before any treatment. HCC diagnosis was made by either 1) biopsy or 2) typical enhancement patterns on dynamic contrast-enhanced CT or MRI (later codified as Li-RADS criteria). Staging performed per Barcelona Clinic Liver Cancer system (BCLC) (0 very early, A early, B intermediate, C advanced, D terminal). Patients with CCA were diagnosed by histopathology of the liver resection. Patients with LC underwent standard clinical surveillance for HCC development with abdominal ultrasound and AFP testing every 6 months (optimally) per AASLD surveillance guidelines. Patient details are shown in **Table 1** and **Supplementary Table 7**. Normal human liver tissue was a kind gift from Dr. Ramilla Philip, Immunotope Inc.

**Table 1:** Clinical and demographic information of patients used as a source of plasma and tumor tissues for RNAseq analyses.

	n	Age	Gendern=M/F	HBV/HCV/NAFl/NASH	LC (n)	HCC stages(n)	HCC Stages(n)
						AJCC I, II or BCLC A, B	AJCC Ill or BCLC C,D
NHC	6	60.6	313	0/0/0	N/A	NIA	N/A
LC	8	64	8/0	3/3/0	8	NIA	N/A
HCC	14	64.1	11113	1/6/2	11	10	4
CCA	2	72.5	1/1	1/0/2	2	1	1
Normal liver	1	57	1/0	0/0/0	N/A	NIA	N/A

Early cancer (AJCC I,II;Barcelona A,B);Late (AJCC Ill;Barcelona C,D).

N/A, Not Applicable.

Plasma from 30 different individuals were used. N=6, from “Normal healthy Controls (NHC), n=8 from those with a diagnosis of Liver Cirrhosis (LC), n=14 from those with a diagnosis of hepatocellular carcinoma (HCC) and LC and n=2 from those with a diagnosis of cholangiocarcinoma (CCA). For the HCC group: 11 of the 14 were also diagnosed with LC, 2 with NAFL; 10 BCLC A, B or AJCC I, II; 4 with BCLC C, D or AJCC III A & B. All patient specimens were acquired at the time diagnosis before initiating any therapeutic treatment. Ages and gender (Male (M), Female (F)), as indicated. Individuals with chronic hepatitis B virus (HBV) or hepatitis C virus (HCV) or no evidence of viral hepatitis were included, as indicated. Diagnosis of HCC, LC and chronic viral hepatitis as in text.

### Isolation and characterization of EVs

Plasma samples from HCC or CCA patients (1 to 1.5ml) were spun at 2,000g for 30 minutes to remove the cellular debris before purification of EVs using ExoQuick kit (System biosciences, Palo Alto CA). EVs were further purified by ultracentrifugation at 100,000g for 2 hours at 4C, pellet resuspended in 100ul PBS. followed by NanoSight tracking and Chip analysis. Dilutions of samples with PBS (Zetaview NTA) were carried out as 1:1000 (HCC001), 1:100 (HCC004, HCC006, HCC007, CCA005, NHC66), 1:20,000 (HCC014) and 1:10,000 (LC19, LC34). Dilutions for Exoview chip analysis were carried out in solution A (Exoview tetraspanin plasma kit analysis) 1:1000 (HCC001),1:100 (HCC004, CCA005), 1:100,000 (LC19). Manufacturer instructions were followed and EVs were stained for CD63, CD81 and CD19 at 1:1000 final working dilutions from the stock concentration. Antibodies against these markers are immobilized on chips called capture probes. Once they encounter sample, tetraspanin receptor proteins on EVs bind to the antibodies on the chip and after washing, the chips are treated with fluorescently labeled antibodies against tetraspanin receptors, just like a sandwich ELISA. After washing, fluorecent labels are detected to evaluate particle counts and relative concentrations of tetraspanin receptors.

### RNA extraction

Total RNA was isolated from human plasma samples, tumor and normal tissues and plasma-derived extracellular vesicles as reported earlier (33).

### RNAseq analysis

For characterizing the circulating transcriptome, as shown in **Table 1**, plasma samples from HCC patients (n=14) representing both early and late stage, LC patients without HCC (n=8) and normal healthy individuals (n=6) were analyzed by RNAseq analysis. Within the HCC patient group, we also investigated matching tumors from 6 patients and plasma derived EVs from 5 patients in parallel. One normal human liver tissue was also included in the study. Total RNA was isolated and RNAseq was carried out on either Illumina NextSeq 500 platform using the SMARTer^®^ Stranded Total RNA-Seq Kit v2-Pico Input Mammalian (Takara #634411) on a highoutput flow cell 2x75 bp as reported earlier (25) or on the Illumina NovaSeq 6000 using a S1 Flow Cell with a paired end run, 2 x 150 cycles, generating more than 50 million reads each. Residual Pico v2 SMART adapters on paired end fastq files were trimmed. The expression of genes in each sample were called with RSEM with STAR (2.7.5a) as aligner against a human reference genome (GRCh38.p13).

### Variant calling and derivation of high-risk variants

Briefly indel local realignment, base quality recalibration and variants calling (Haplotype caller algorithm) were performed using Broad Institutes Genome Analysis Tool Kit (GATK) version 4.1.7.0. Variants with 20 or higher quality score as well as depth of 5 or more were considered as true positive. Filtration and annotation of high-risk variants was carried out using SnpEff analysis (version 4.3t) to identify high risk variants in all sample subsets. The SNPeff uses sequence and structure-based bioinformatics tools to predict the effect of protein-coding SNPs on the structural phenotype of proteins. It includes intronic, untranslated region, upstream, downstream, splice site, or intergenic regions in its annotated genomic locations. It can predict coding effects such as synonymous or non-synonymous amino acid replacement, start/stop codon gains or losses, or frame shift changes. Tumor-centric high-risk variants were sorted by concordant detection in tumors and plasma from HCC patients, proportion/frequency in samples, lack of detection in normal liver tissue and in the plasma of LC and NHC controls and established functional association with cancer. Pathway enrichment analysis was carried out using R package ClusterProfiler.

### Statistics

The distinguishing high-risk variant transcripts were selected out of 5,480 tumor specific variants using Fisher’s exact test with multiple testing adjustment *via* Benjamini–Hochberg method.

## Results

### Diversity of RNA variants and variant effects

**Figure 1:**
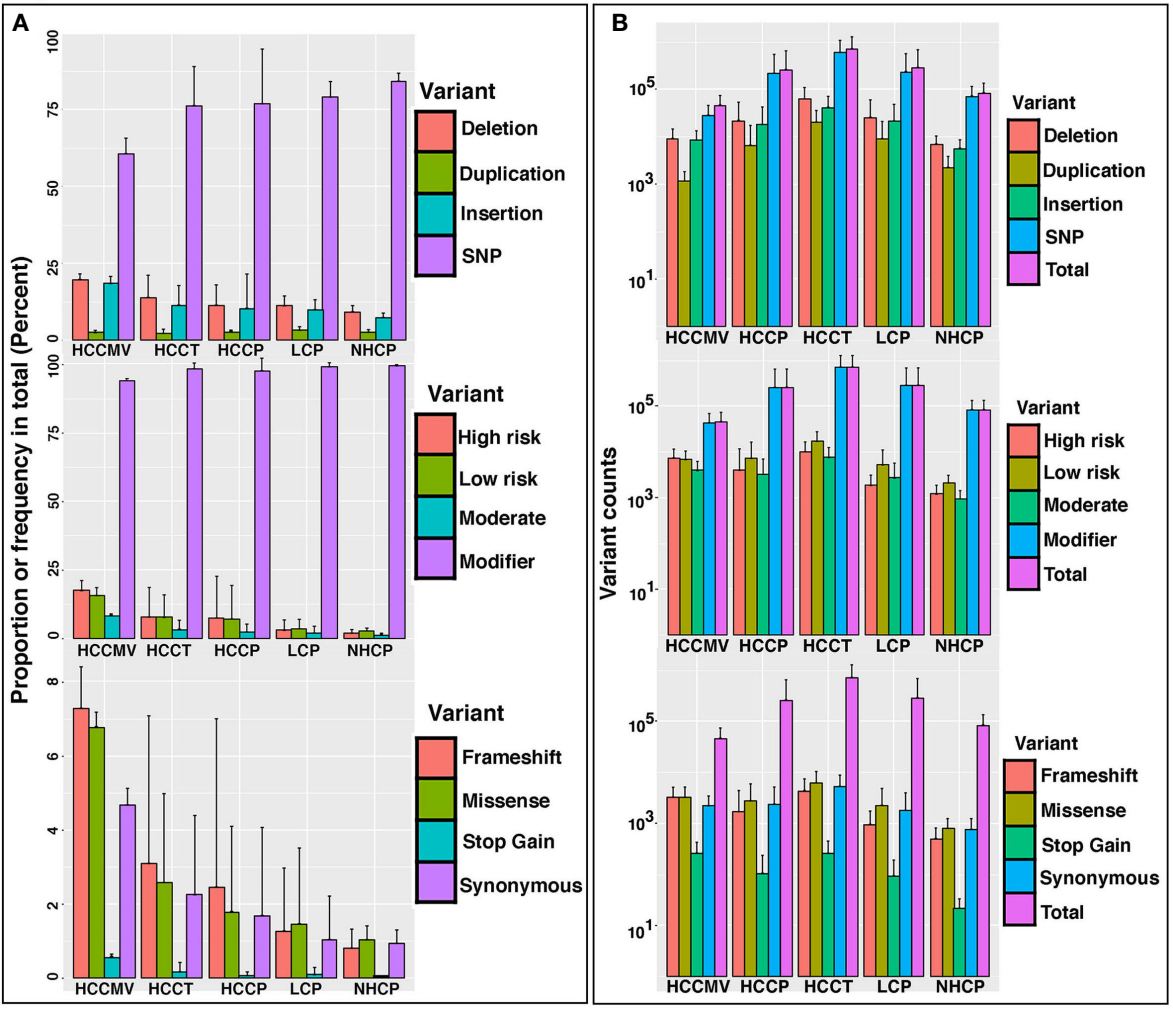
Diversity and profiles of circulating mRNA variants detected in the circulation, circulating extracellular vesicles and tumors of HCC patients. **(A)**. Bar charts representing proportion or frequency of occurrence (% age) in patient and control specimens; HCCMV, HCC circulating microvesicles (n=6); HCCT, HCC tumors (n=6); HCCP, HCC plasma (n=14); LC, liver cirrhosis plasma (n=8); NHCP, healthy control plasma (n=6). Separate bar graphs are shown to represent profiles by type, impact, and functional class of variants. Error bars represent the variation within samples. **(B)**. Bar graphs representing the total number and number of variant counts corresponding to different categories in the same sample subsets as 1A. The average number of variants per sample in RNA collected from different sources of subjects represented by the height of the bar, while the error bar showing the standard deviation within the group. Variant calling used a depth >=5 and QUAL value >=20. The ‘QUAL’ value for each called SNP is calculated as -log10(p) where p is the p value yielded from the statistical test carried out by ExactSNP. Therefore, a ‘QUAL’ value of 20 corresponds to a p value of 10^-20, which is extremely low.

Bioinformatic tools help determine the effect of variants (SNPs, indels, CNVs, structural variants) on genes, transcripts, protein sequences and regulatory regions. RNAseq variant analysis in 46 samples (Tumors, plasma, circulating EVs and normal liver tissue from 29 subjects) led to the detection of some 27.5M variant effects manifested due to 5.2 M SNPs, 0.91M insertions and 0.81 M deletions. Measuring by impact, 94.3% represented modifier variants, followed by high and low risk variants at 2.3% each and 1% of variants were of moderate risk. Among the effects by functional class, 62% represented missense variants, synonymous/silent 35% and stop gain/non-sense variants at 3%. Evaluating effects by type and region, 56% were detected in introns, upstream 12.5%, downstream 12.6%, intergenic 10.5%, exon 4.2%, splicing 2.5% and 0.05% are genic. No significant differences were observed when comparing these effects between sample groups. However, proportion of high and moderate risk, frame shift and missense variants was observed to increase as we moved from normal healthy, LC to HCC patients. Though the increase in sample subsets was not statistically significant, it did point to a possibility of progressive build-up of mutations during disease progression (**Figure 1A**). **Figure 1B** shows the average counts of variants corresponding to different categories in various clinical subsets.

### Tumor-centric concordant variants

**Figure 2:**
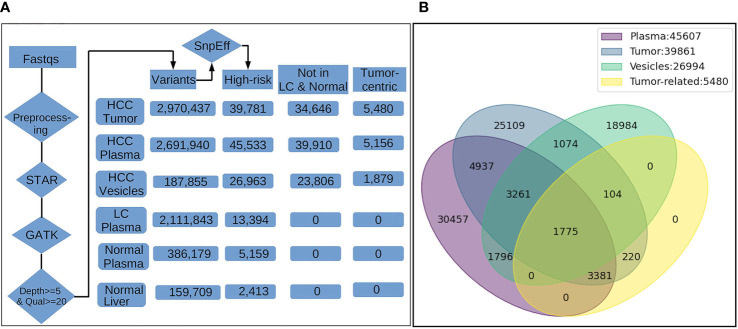
Filtration summary for derivation of high-risk HCC-specific mRNA variants and concordance of detection between different sample subsets from HCC patients. **(A)**. The FASTQ files from NGS platform (illumina) were pre-processed to remove the residual of sequencing adapter in the files. The trimmed data were input into STAR, an aligner for RNA-Seq mapping, for alignment against human reference genome GRCh38. The alignments (BAM) were processed with GATK tools (v4.1.7.0) for spliting on reads with N cigar, base quality score recalibration, and variants calling by HaplotypeCaller. The variants reported by GTAK were filtered by depth (>=5) and quality (>=20) and annotated with snpEff for location on genes/transcripts, mutation type and putative biological impact. The number in the boxes indicate the total unique variants identified in samples of different groups. Variants occurred in multiple samples within the same group were counted once only. Only high-risk variants and those with highest frequency in tumors and HCC plasma were considered during enrichment. High-risk variants also detected in the circulation of liver cirrhosis patients and normal healthy individuals were excluded leading us to cancer-associated high-risk variants referred to as tumor-centric variants. **(B)**. Venn diagram showing the distribution and overlap of tumor-centric variants with total high-risk variants in various sample sources. The number of High-risk variants identified only in a single sample type either plasma, tumor and vesicles are 30,457, 25,109 and 18,894, repectively, reflecting the heterogeneity and difference in variant profiles attributed to the source of RNA. However, there are still 5,036 variants occurring in RNA from all three sources. Furthermore, 1,775 of them were among 5,480 tumor-related variants identified by tumor-control comparison, suggesting the plasma/vesicle RNA share abundant/significant transcripts carrying the tumor associated variants.

We then evaluated the occurrence of “high risk” variants in each category. Filtration and annotation of high-risk variants was carried out using SnpEff analysis to identify high risk variants in all sample subsets (**Figure 2A**). “High risk” variants, derived by SnpEff are predicted to cause a significant effect on protein structure. As mentioned in methods, the SnpEff uses sequence and structure-based bioinformatics tools to predict the effect of protein-coding SNPs on the structural phenotype of proteins. High-risk variants in HCC tumors were further filtered to include variants detected in the plasma of HCC patients (concordant variants) and remove any variants detected in normal liver tissue and plasma samples of LC patients and NHC controls to derive a set of circulating tumor-centric RNA (ctRNA) variants. Analysis of 14 plasma and 6 corresponding tumors from HCC patients identified 5480 high-risk concordant ctRNA variants representing 3199 genes and detected in high frequency in both circulating plasma (7-50%) and tumors (30-100%). None of these tumor-centric variants are detected in the plasma of either LC patients (n=8) or NHC (n=6) controls. Most of the high-risk concordant variants involve splice donor/acceptor regions (4933), frameshift (2506) changes and splicing variations. Comparing tumor centric variants with all SnpEff derived high-risk variants identified in HCCT, HCCP and HCCMV sample subsets point to the concordant variants shared between the different sample subsets (**Figure 2B**).

### Prominent altered genes corresponding to variant transcripts.

**Figure 3:**
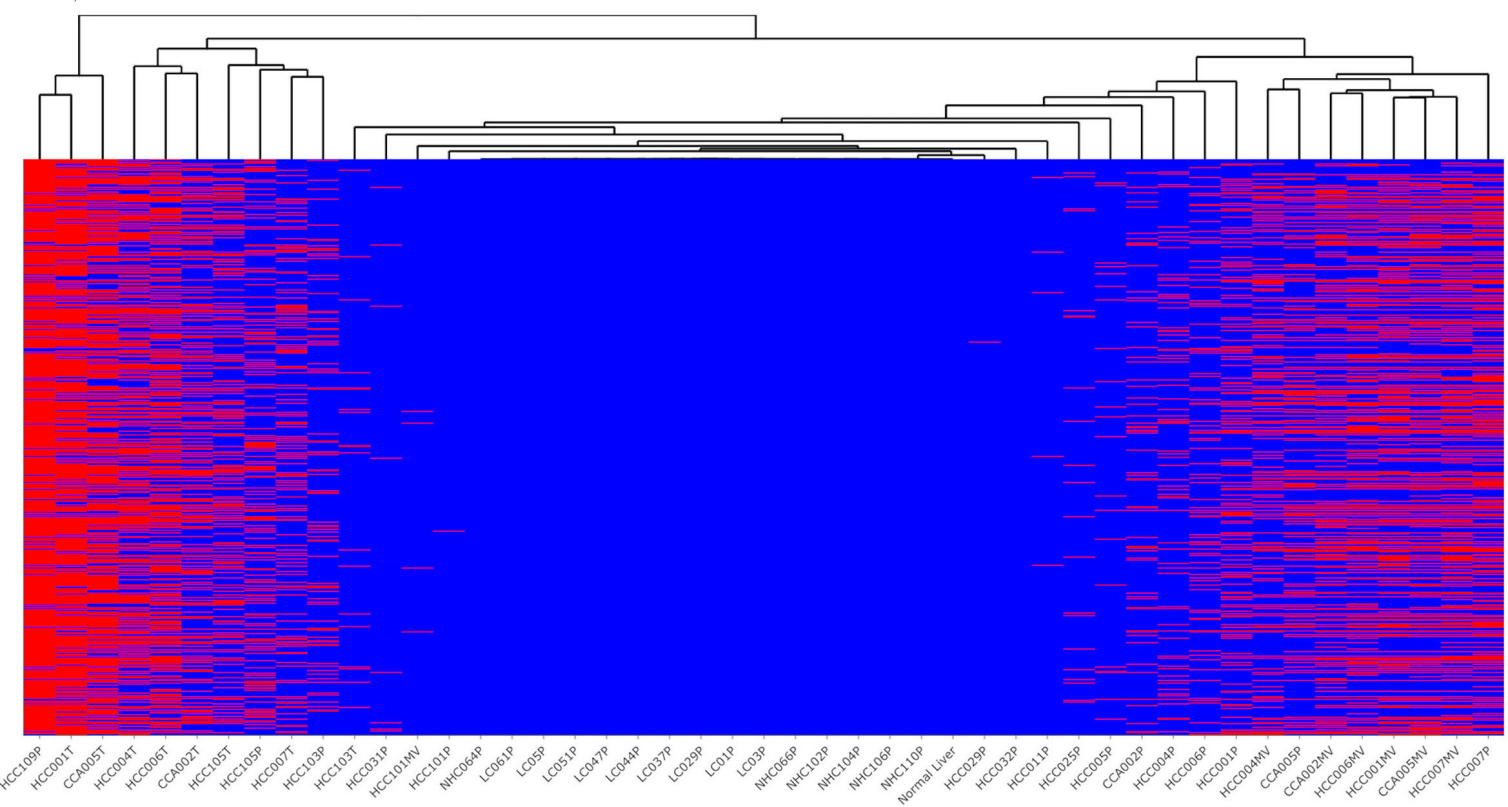
Profile of top high-risk concordant variants detected selectively in the plasma and tumors of HCC patients. Indicated occurrence (red color) of 1,501 high-risk variants identified in plasma of HCC patients but not in liver cirrhosis and healthy controls, with clustering dendrogram of samples on the top. Most of the tumor samples were clustered on the left part of the figure, while the vesicle samples on the right side. The liver cirrhosis and healthy controls not carrying the variants (blue) stay in the middle section. The variant annotations and profiles of these variants is shown in **Supplementary Table 1**.

**Figure 3** represents ctRNA variants detected in tumors and circulation of HCC patients in a clustering heat map comprised of splicing variants, SNPs, frameshift variants etc. (**Supplementary Table 1**). Notable high-risk variants present in tumors and plasma (concordant) correspond to TP53, CTNNB1, FAH and SF3B1 genes, which are already known to harbor driver mutations (reviewed in 39). Concordant variant transcripts corresponding to complement cascade like C2, C4A, C4B, C1R, C5, C8G, KLKB1, KNG1, CFHR5, MASP2, etc. which are known to be liver enriched (40) are represented among tumor-centric ctRNA variants. Additionally, among tumor-centric variants figure those ctRNA variants corresponding to coagulation factors F2, F11 and F12, which are not only liver enriched but also represent FDA approved drug targets (40).

### Liver-derived transcripts reveal a high frequency of variants

**Figure 4:**
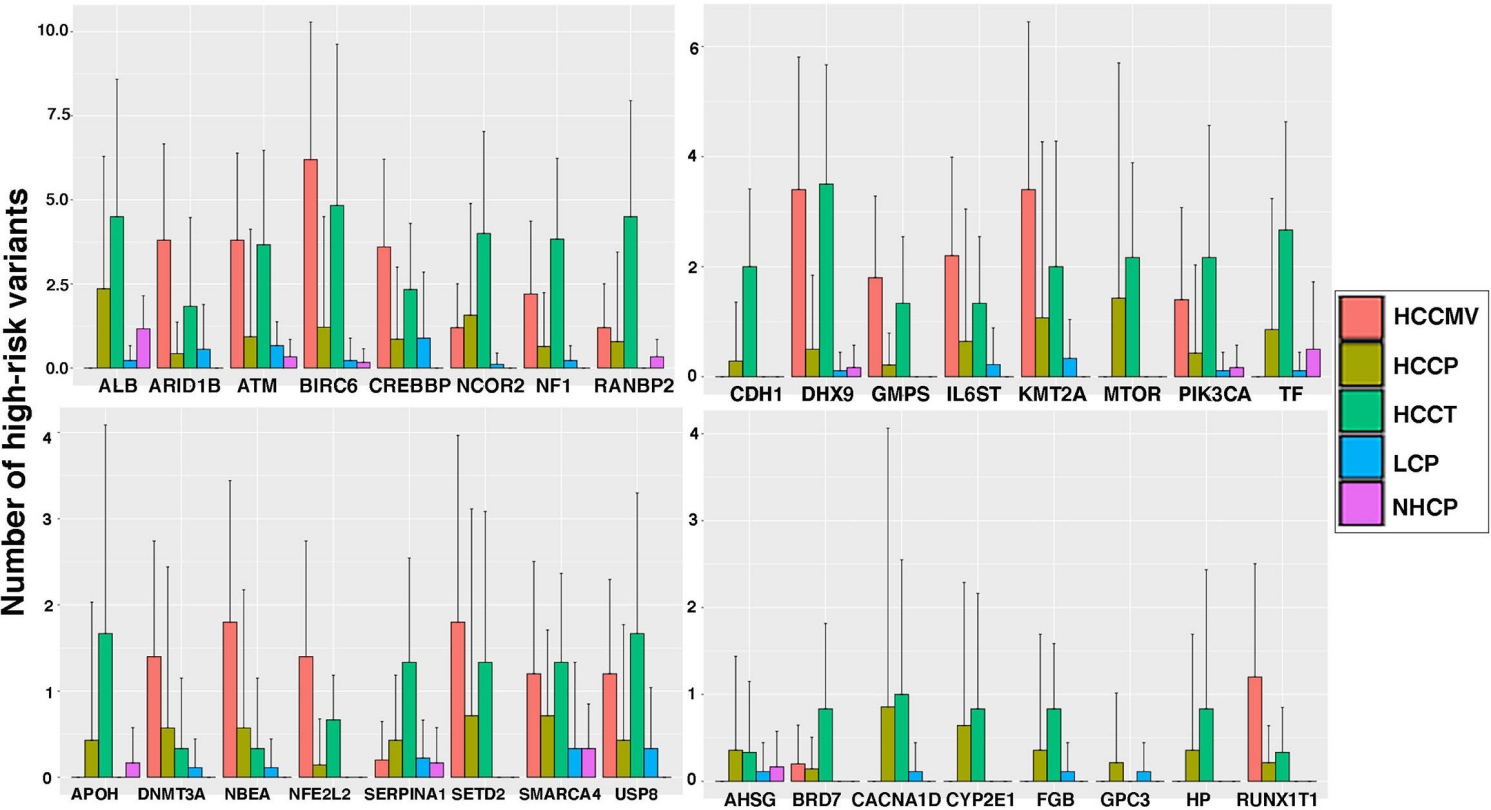
Liver specific transcripts in HCC patients reflect higher levels of high-risk variants than those from liver cirrhosis patients and healthy controls. The bar graphs represent the average counts of high-risk variants on 32 liver specific transcripts in several sample groups and error bar showing the standard deviation. Clearly plasma from HCC patients show significantly higher mutation load than that of LC patients.

With an interest to understand the variants in liver-associated transcripts, we calculated the number of total high-risk variants detected on these transcripts in various patient subsets. Some well-known liver-derived transcripts like FGL1, CYP2E1, SERPINA1, ALB, TF and SYCE1 display a huge variant load, particularly in the plasma and tumors from HCC patients. Several other liver-associated transcripts were observed to harbor significantly higher numbers of unique, high-risk variants in tumors, plasma or EVs of cancer patients compared to the levels detected in circulating plasma from LC patients and NHC controls. Bar graphs representing the total number of high-risk variants present in liver-associated transcripts in various clinical subsets are shown in **Figure 4**. Focusing on certain selective tissue specific transcripts with associated high-risk variants can potentially offer increased specificity and sensitivity to identify high-risk patients.

### Characterization of extracellular vesicles (EVs) isolated from plasma

**Figure 5:**
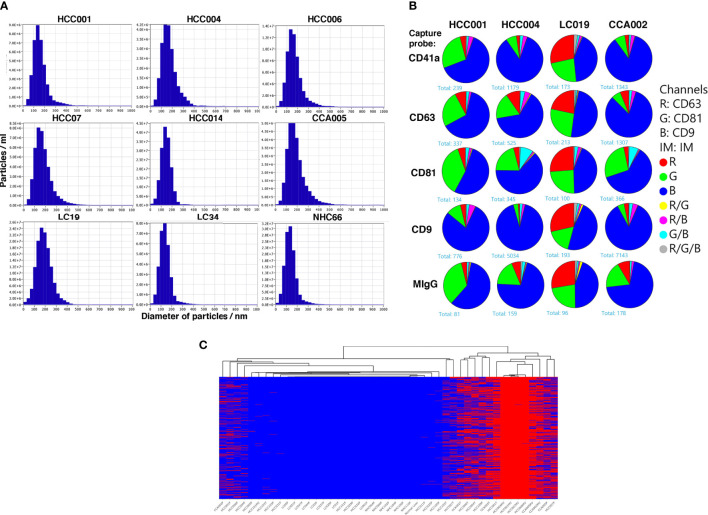
Characterization of extracellular vesicles isolated from the plasma and profiles of high-risk tumor specific mRNA variants within the EVs of HCC patients. **(A)**. Plasma samples from HCC patients, LC patients without HCC and normal healthy controls were spun to remove the cellular debris before precipitation of EVs using ExoQuick kit followed by ultracentrifugation. followed by NanoSight tracking and Chip analysis. Dilutions of samples with PBS were carried out as shown in methods followed by Zetaview NTA analysis. 11 positions/frames were used to analyze each sample. Y-axes represent particles/ml and x-axes represent particle diameter in nm. The highest value on y axes reflects the number of particles/ml at the peak. **(B)** Exoview chip analysis was carried out to evaluate tetraspanin receptor expression. EVs stained for CD63, CD81 and CD19 antibodies and anti CD41a and mIgG used as controls. Data is represented as pie charts to reflect the tetraspanin receptor profiles in EVs from two HCC, 1 LC and 1 CCA patients. LC patient derived EVs seem to have a higher expression of CD63 compared to HCC or CCA patients. **(C)** Heat map representing occurrence (red color) of 1,369 high-risk variants identified in EVs of HCC patients but not in LC and NHC controls, with clustering dendrogram of samples on the top. Most of the EV samples are clustered on the right side, along with RNA from cancer patients (tissue and plasma). Annotation of variants is shown in **Supplementary Table 2**.

It was interesting to know if the variant mRNA transcripts were present in the circulation within extracellular vesicles (EVs) since (EVs) in circulation are known to contain nucleic acids. Therefore, in five HCC subjects we compared the variant mRNA profiles in whole plasma with matching plasma derived EVs by RNAseq. EVs were purified from HCC, HCC-free LC or NHC plasma samples and analyzed for size and density by zeta view (Ncsi Spectradyne) analysis. **Figure 5A** shows the density peaks of circulating EVs from different patients and healthy control all centered around a 150 nm size. The EVs were further characterized for expression of tetraspanin receptors using Exoview chip arrays (**Figure 5B**). Pie charts represent the expression profiles of CD9, CD81 and CD63 in percentage of circulating EV particles from representative cancer patients and LC control. CD41a is a platelet marker, antibody against which was used together with IgG control. Data shows that all EVs seem to reflect comparable expression of the tetraspanin receptors authenticating their EV phenotype.

#### Common concordant variants selectively enriched in circulating EVs of HCC patients

RNAseq analyses of circulating EVs from five HCC patients were performed and the results were compared with RNAseq from their corresponding tumors. A set of concordant variants selectively enriched in EVs of these patients are highlighted (**Figure 5C**; **Supplementary Table 2**). Briefly, as shown in **Figure 5C**, 218 high-risk concordant variants detected in tumors and plasma derived EVs of HCC patients were identified. Variants identified in this category correspond to some known HCC related transcripts like GOLGA2, ASAH1, ODC2, SAR1A, PIK3R1, CCNG1, MACF1, SERPINE1, CTNNB1, CREB3 and ADH5, among others. We have not investigated and compared these variants to the EVs from LC or NHC samples. However, none of these EV-enriched variants are detected in the plasma of NHC controls or LC patients. The data indicates unique EV enrichment of these circulating variants.

#### Analyzing matching samples, whole plasma, circulating EVs and tumors from same cancer subjects indicated triple-concordant high-risk variants

**Figure 6:**
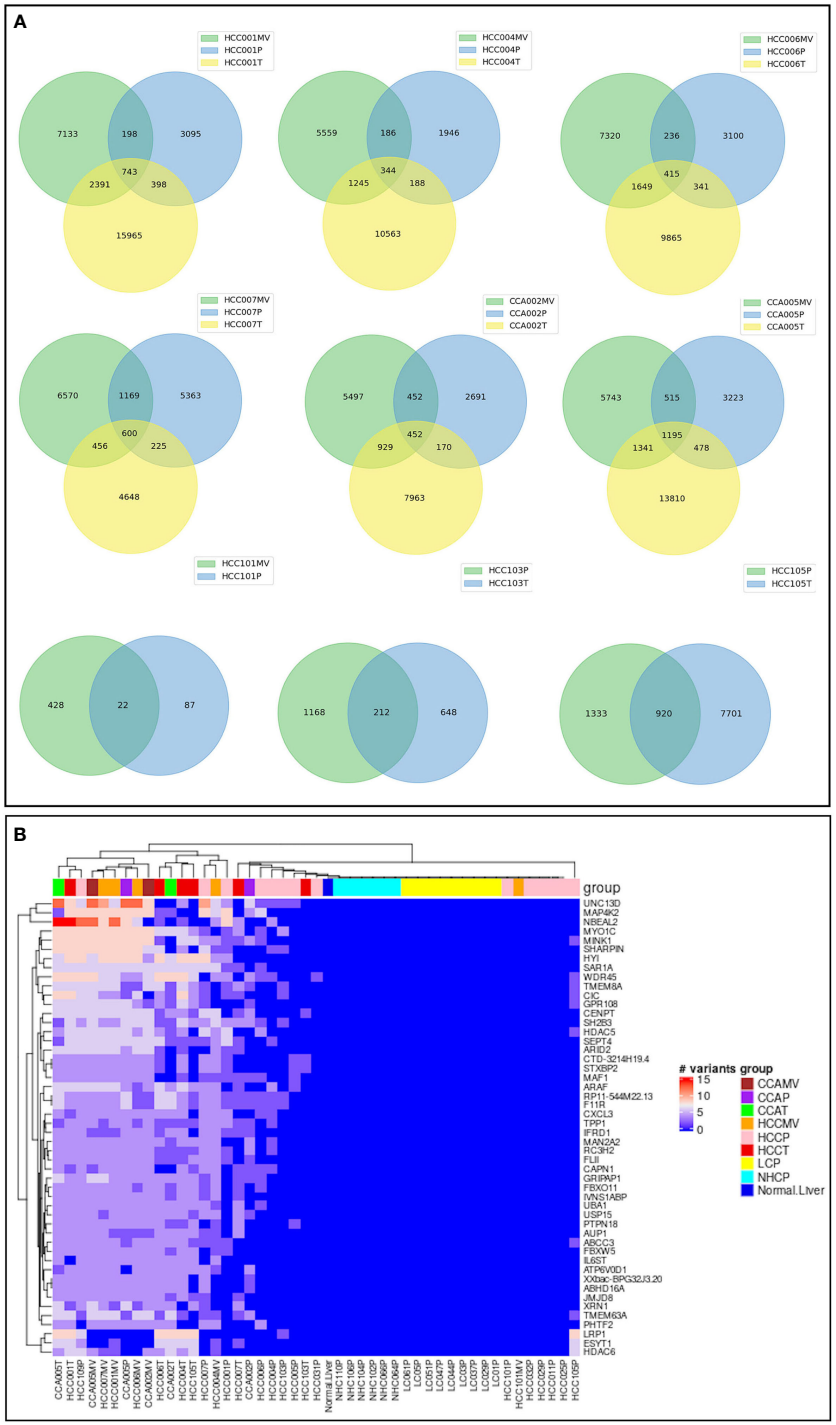
Concordance of high-risk variants in three matching samples from same cancer patients. **(A)**. Venn diagrams corresponding to 6 cancer patients (4 HCC, 2 CCA) with three matching specimens, EVs, tumor and plasma from each subject and three subjects with two matching specimens. The numbers represent the distribution and concordance of high-risk mRNA variants in multiple samples from same patients. **(B)**. Heat map representation of top genes carrying highest number of high-risk concordant variants. The number of high-risk variants, which were identified simultaneously in different types of samples (plasma, tumor, and/or vesicle) from same cancer patient, but not presented in any of liver cirrhosis or healthy control, were summarized by gene carrying them. The first 50 genes carrying the most of such high-risk concordant variants were selected and the numbers of variants on those genes were plotted against samples. UNC13D shown at the top, for example has the highest number of variants in HCC patient specimens. Annotation and profiles of these variants can be found in the **Supplementary Table 3**.

Significantly, comparing three different specimens from the same cancer subjects identified a set of triple concordant variants not detected in the circulation of LC patients or normal healthy controls. Hundreds of such triple concordant variants were detected in a set of 8 cancer patients with matched specimens (**Figure 6A**). Venn diagrams show the distribution of these variants in matching specimens from same subjects and strongly suggest an association of these high-risk variants with cancer. We sorted triple concordant high-risk variants from all patients and organized the corresponding genes based on number of variants (**Figure 6B**; **Supplementary Table 3**). The heat map in **Figure 6B** represents the distribution of top genes sorted based on high number of high-risk triple concordant variants. The data highlight the top altered genes mutated frequently at several critical regions and underscores the authenticity and functional relevance of these ctRNA variants. For example, UNC13D is a calcium dependent cytoplasmic protein involved in vesicular and endocytic transport essential for vesicle maturation and docking and also known as an unfavourable prognostic marker in renal, endometrial and pancreatic cancer (40). Nurobeachin like 2 (NBEAL2) plays a role in development and secretion of alpha granules containing growth factors and is a prognostic marker for head and neck cancer (40). MAP4K2 is a serine/threonine protein kinase and an essential component of MAP kinase signaling. It acts as an upstream activator of stress activated protein kinase/c-jun N-terminal kinase (SAP/JNK) and p38 pathways. MAP4K2 can be activated by TNFα and interacts with TRAF2. MAP4K2 is known as an unfavourable prognostic marker of liver cancer (40). Presence of multiple high-risk variants on these transcripts reflected in all three specimens from same cancer subjects and lack of detection in non-cancer control subjects highlights their role in cancer pathology.

### Variants associated with CCA

**Figure 7:**
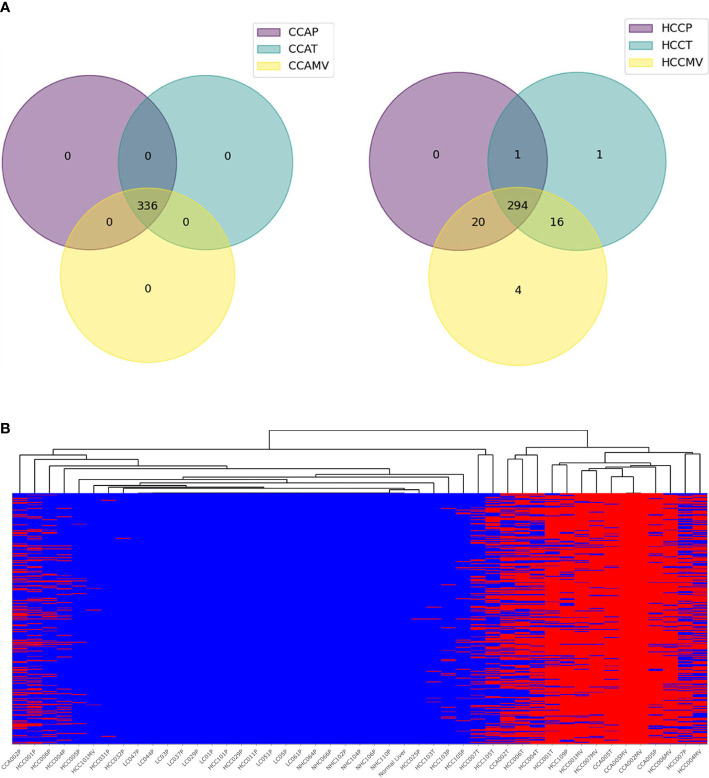
High-risk concordant variants associated with cholangiocarcinoma. **(A)**. Pie charts representing profound synergy in high-risk variants between HCC and CCA patients. All 336 high-risk variants concordant in CCA samples (n=6) are also detected in HCC samples but none in LC and NHC controls. **(B)**. Heat map representing the profiles of concordant CCA variants in various samples. The annotation of variants is shown in **Supplementary Table 4**.

Cholangiocarcinoma is a liver cancer of cholangiocytes, and not hepatocytes. It was therefore of interest and opportunistic to examine a set of specimens of matched plasma and tumor for high-risk mRNA transcripts. Matched CCA plasma, CCA EVs and tumors from CCA patients were studied as additional liver cancer controls to test the specificity of HCC-specific variants. Surprisingly, most of the highly frequent high-risk variants in HCC patients were also detected in CCA samples. We sorted variants on the basis of their proportion in CCA patients (**Figure 7**; **Supplementary Table 4**). As in HCC, detection of high-risk variants in CCA patients was more frequent in tumors than plasma samples. EV-enriched HCC associated variants were also detected in the EVs of CCA patients. This concordance of ctRNA variations between HCC and CCA is interesting and underscores the functional relevance of such variants and validates their specificity in liver cancer.

#### Variants corresponding to transcripts involved in cancer and metabolic pathways suggest widespread metabolic alterations in HCC

The high-risk mRNA variants we have identified are selectively present in the people with HCC. Given that the genes corresponding to these variants are well known players in tumor biology we hypothesize that these variants reflect the mutational environment of the tumor cells, from which we believe they are derived. To emphasize the importance of mutations in cancer biology, a multi-analyte blood test named as CancerSEEK based on circulating proteins and cfDNA mutations has been reported to detect 8 common cancers (41). Malignancies including HCC are characterized by the presence of mutated genes, possibly a result of altered tumor metabolism, DNA repair and splicing and our unique finding is that these genetic lesions can be detected in circulating transcripts.

**Figure 8:**
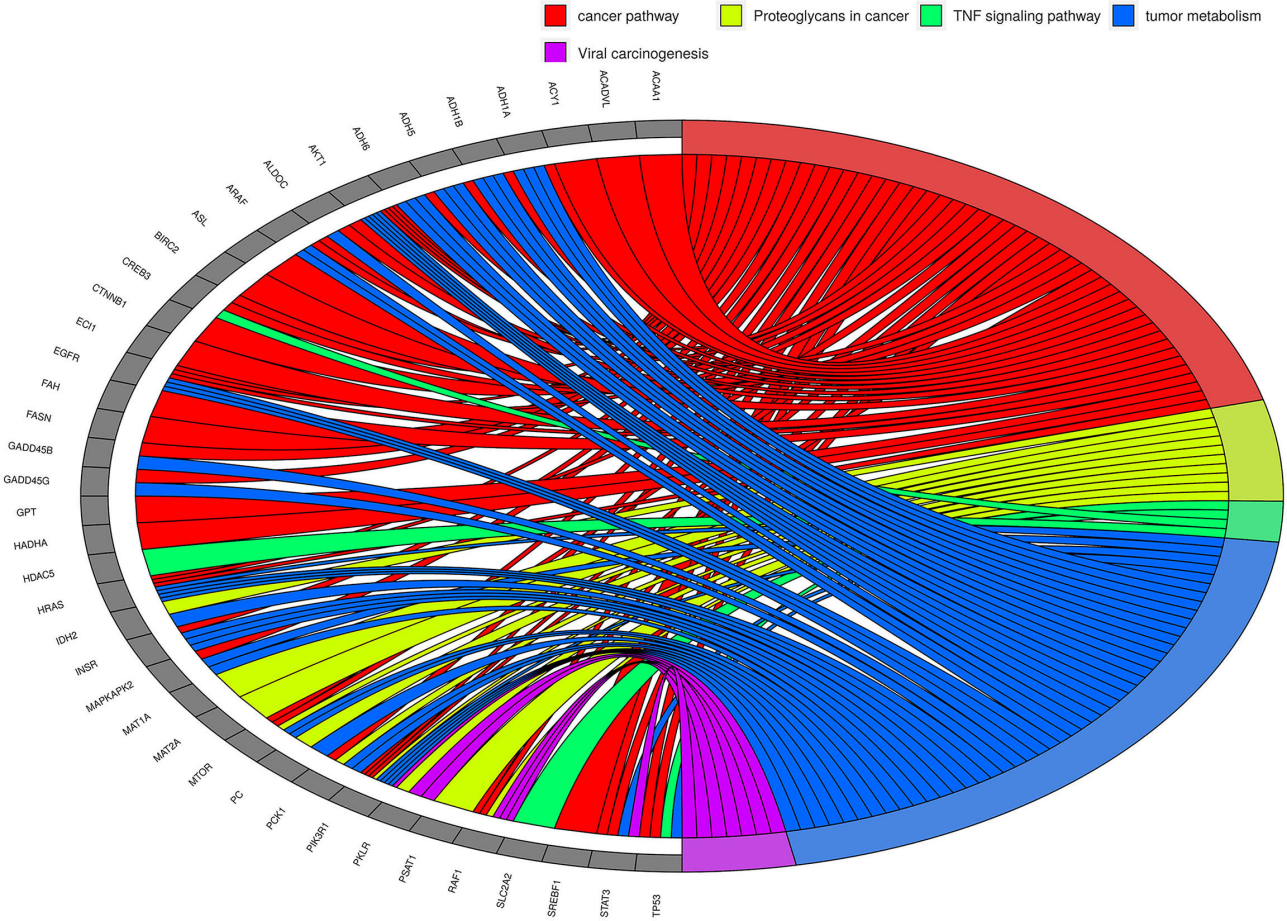
The KEGG pathway/Gene Ontology enrichment analyses on top tumor-centric variants. Transcripts carrying tumor-related mutations show clustering of these genes in cancer-related pathways and biological processes. Chord in the diagram indicate the connection between genes (grey blocks at left side) and pathways/processes (colored blocks on the right side). Some pathways/processes were merged for simplification. As can be seen, metabolism-related genes seem to be predominantly altered in HCC.

Though we have not characterized any NGS derived concordant tumor-centric variants, high-risk variants identified in HCC patients using GATK and SnpEff tools correspond to transcripts of EGFR, CTNNA1, PTPA, AKT1, MTOR, ARAF, RAF1, HRAS, CREB3, SREBF1, ACY1, STAT3, etc. These genes correspond to well-known cancer pathways. We carried out KEGG pathway enrichment analysis on 578 genes corresponding to tumor-centric variants (**Supplementary Figure 1**) and were able to identify major pathways corresponding to variant genes. A profound and predominant representation of circulating tumor-centric variants correspond to genes involved in metabolism suggesting some interplay in altered tumor metabolism (**Figure 8**). Concordant variants of FASN, PC, SREBF1, ACADVL, PKLR, PCK1, ALDOC, PSAT1, MAT1A, MAT2A, GPT, etc. point to the altered genes associated with dysregulated tumor metabolism. In comparison, none of these tumor-centric variants were detected in the circulation of LC or NHC subjects. Variants corresponding to several other liver enriched transcripts like IDH2, PROC, PKLR, ACAA1, KLKB1, ACY1, ASL, MASP2, ECI1, ADH1A, ADH1B, ADH6, HADHA, etc. are well known metabolic enzymes facilitating tumor adaptation and some of these transcripts represent potential or FDA approved drug targets. Variants of GPT and PSAT1 are liver enriched and play role in glutamine metabolism and signaling. Variant transcripts associated with insulin signaling pathways (40) like PIK3R1, SLC2A2, SLC27A, PTPRF, OGT, NR1H, etc. are among the concordant ctRNA variants detected in both plasma and tumors of HCC patients.

The preliminary data are exciting and provocative. Though the number of samples analyzed is small, the data illustrate a high mutation milieu in HCC patients and highlight the diagnostic potential of circulating HCC-specific mRNA variants detectable in a non-invasive manner.

## Discussion

Detection of tumor-derived genetic material from peripheral blood offers a non-invasive platform with an additional advantage that a full spectrum of mutations in tumors can be detected compared to tumor biopsies that may miss variants not uniformly distributed due to tumor/nodular heterogeneity (16). Mutations are ubiquitous in cancer and the association between somatic mutations and cancer is well recognized (42). The inflammatory environment in hepatitis, MAFLD and cirrhosis may serve as a premalignant stage, the development of HCC requires progressive pre-neoplastic changes and further disease progression leads to enhanced tumor heterogeneity and increased mutation load (43, 44). Mutational signature is known to be largely conserved across the liver (45, 46).

An increasing number of studies demonstrate the potential use of cfDNA in diagnosis, prognosis, and monitoring of cancer. A multi-cancer early detection CancerSeek blood test based on specific DNA mutations and protein biomarkers when combined with PET-CT has shown progress in early detection (47). HCCscreen test (Genetron) claims detection of early-stage HCC with high sensitivity based on DNA mutations and protein biomarkers (48). Mutations within a tumor can be clonal or subclonal, and the amount of available genome copies is a limiting factor for the detection of variants of low allele frequency (49, 50). Moreover, the tumor fraction of circulating DNA (ctDNA) varies between cancer types as well as between patients affected by the same cancer type (40). Even at the metastatic stage, some patients can yield a low amount of ctDNA (51, 52). Challenges in the optimization and standardization of preanalytical steps, significant gaps in our understanding of its origin, physical properties, and dynamics in circulation call for alternative approaches which can address and overcome low allelic frequency and limited copy number of target variants (24, 27, 53, 54).

The presence of mRNA sequences in the circulation has begun to be recognized (55–58). Our laboratory is the first to demonstrate mRNA as a powerful analyte with significant upregulation of HCC-specific transcripts in the circulation of HCC patients compared to high-risk patients without cancer (33). Another study using circulating EVs reported a ten-gene RNA expression signature, consistent with our data, exhibiting high sensitivity in distinguishing early-stage HCC from LC patients (59). We now provide evidence of specific high-risk mutations in tumor-derived liver transcripts in the circulation of HCC patients (60) and identify a panel of 9 ctmutRNA with high diagnostic performance (data not shown). Studies on circulating RNA (ctRNA) is an emerging field in diagnostics (57, 58) and transcriptomics-based biomarkers are reported to exhibit higher efficiency than protein biomarkers of HCC (61).

**Figure 9:**
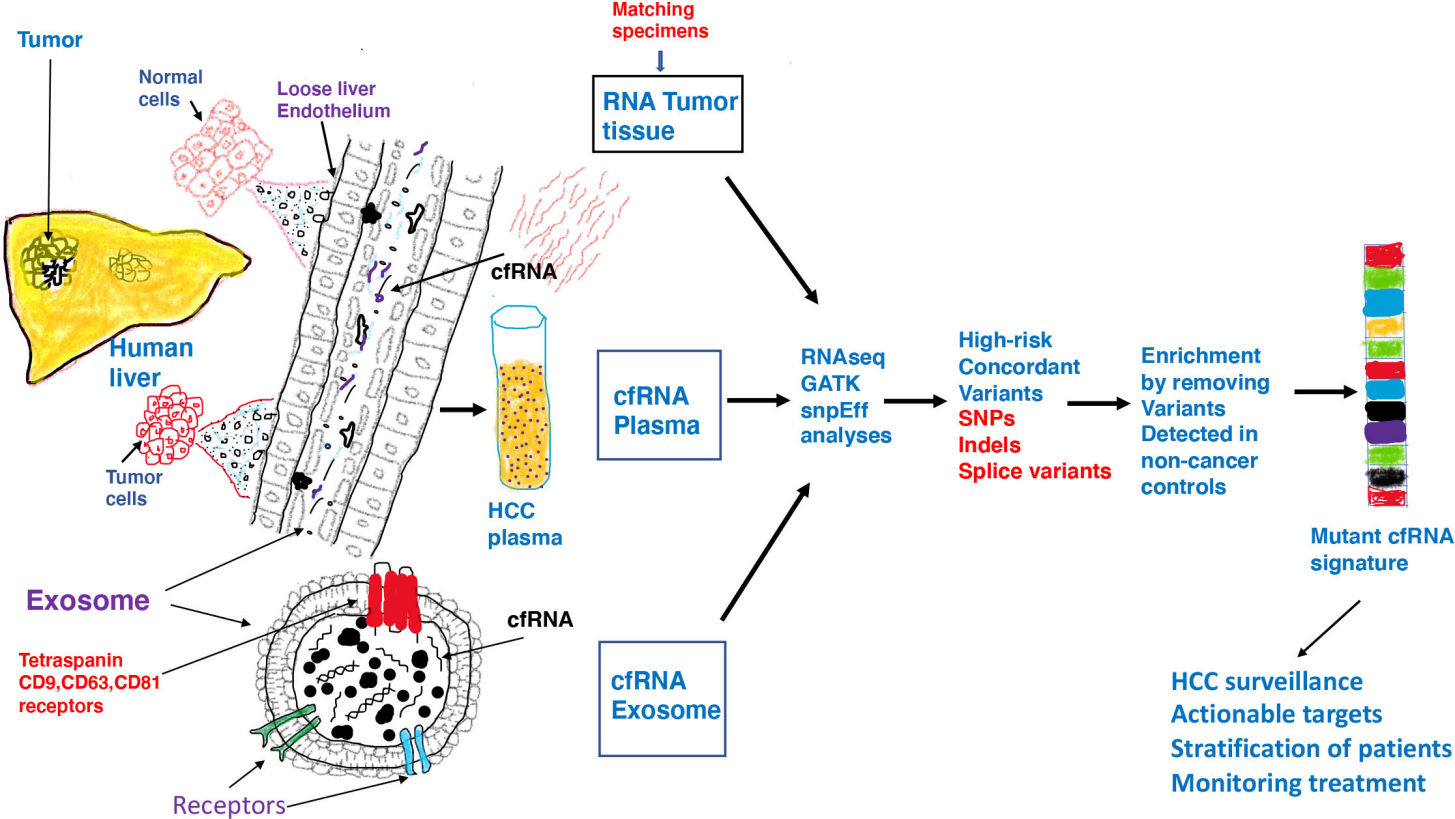
Inflammatory and toxic milieu in the liver influences the tumor microenvironment resulting in release of tumor derived transcripts in the circulation. Malignant tumor cells exhibit pervasive changes in DNA which consequently lead to a variety of changes in gene expression or genomic instability. Both functionally normal and tumor cells in liver communicate with their surroundings through exosomes which are able to enter the loose endothelial vasculature in liver and land in the circulation. In addition to DNA, proteins and lipids, both free floating RNA and RNA within exosomes are found in circulation. Exosomes are vesicles of endosomal origin with tetraspanin receptors embedded in the protein lipid bilayer. Exosomes represent safe vehicles to protect nucleic acids and other cargo from degradation in circulation. We investigated RNA from three different samples from HCC/CCA patients by RNAseq, GATK and SNPeff analyses and identified high-risk RNA variants concordant in all cancer samples but not detected in non-cancer liver cirrhosis patients and normal healthy controls. We speculate that after characterization in a larger patient cohort, a mutant RNA signature can be derived which can be validated and used in HCC surveillance to accurately identify high risk patients and actionable targets and guide in patient management.

Here, in this report, by profiling the mutational landscape of the tumor in parallel with that of circulation from HCC patients we identify some top highly frequent 1500 circulating high-risk mRNA variants concordant in tumors and plasma but not detected in the plasma of liver cirrhosis patients or normal healthy controls (**Figure 9**). GATK and SNPeff tools lead to the identity of high-risk variants comprised of both SNP’s and indels most of which seem to be reported in catalogue of somatic mutations in cancer (COSMIC) and dbSNP data bases (**Supplementary Table 6**). Several high-risk variants correspond to cancer driver genes and cancer signaling pathways. Some of these variants correspond to genes known as actionable targets with either potential or FDA approved therapies. Significantly, a large subset of HCC-specific high-risk variants were also detectable in the tumors, EVs and plasma of few cholangiocarcinoma patients but not in LC or normal healthy individuals.

Splicing variants represent a predominant category of high-risk mRNA variants detected in tumors, EVs and free plasma of cancer patients; none of these are detected in normal liver or plasma of LC patients and normal healthy individuals. Alternative isoforms and tumor-specific isoforms that arise from aberrant splicing during the liver tumorigenesis have been reported earlier (62) and long read RNA sequencing in liver cells and tumors have identified spliced variants corresponding to enzyme regulators, chromatin modifiers, RNA-binding proteins and receptors (63). A high level of differential splicing is known to occur in primary HCC tumor tissues compared with normal liver, and many of these changes have been shown to correlate with patient survival (64, 65). It is to be noted that any pathological changes associated with transcription and RNA splicing/editing aberrations cannot be identified through ctDNA. Splicing and fusion variants in ctmRNA together with point mutations could be potentially more effective in identifying early stage and high-risk patients.

We demonstrate that plasma derived extracellular vesicles (EV) are enriched for a subset of high-risk cancer associated mRNA variants which were also detected in matching tumors. EVs provide a mechanism of how tumor-derived variants land in the circulation, are protected from ubiquitous nucleases and variant enrichment provides better chances of detection in circulation. Significantly, overall high-risk variant density was highest in tumors, followed by EVs and lower in free plasma. This suggests that EVs might work as better tools as repositories of mRNA variants compared to free plasma.

Profiling the mutational landscape of the liver through circulating RNA in patients would be a paradigm shifting approach and potentially provide information about tumor biology, patient prognosis and likely response to therapy. Circulating variants of mRNA can offer a better understanding of genetic heterogeneity and if some constitutional variants predispose individuals to a specific molecular subtype of liver tumors. It could enable us to evaluate the exact function of the gene, if not already known, and the consequence of its loss of function in the liver. With regard to the efforts towards precision medicine in HCC, treatment decisions will become increasingly dependent upon genetic stratification and ctRNA variant profiles associated with different etiologies of chronic liver diseases could be very useful at the bedside. The identification of oncogenic activation of tyrosine kinases in some advanced NSCLC tumors, most notably mutations in the *EGFR*, *ALK* or *ROS1* gene, has led to a paradigm shift and the development of specific molecular treatments for patients (66). Moreover, any pathological changes associated with transcription and RNA editing aberrations cannot be identified through DNA.It is becoming apparent that tumor biology (67) and treatment responses, even for immunotherapy (68), may be predictable based on mutational subtypes. Non-invasive and cost-effective access to these circulating mRNA variants could potentially revolutionize clinical management.

Since the fidelity of circulating RNA will be critical for clinical decision making, optimization and standardization of clinical sample collection, RNA isolation and analyses are critical for development of this platform. Our data clearly demonstrate the possibility of this platform to be used for clinical translation. Specific liver associated ctRNA biomarkers could potentially identify patients with liver cirrhosis, MAFLD and malignant pathology. Samples could be easily drawn linearly from patients and repeated for confirmation, at various time points of surveillance and treatment and treatment decisions could be tailored to mutational load of HCC-specific transcripts.

Though identification of these ctmRNA variants in HCC patients was carried out using next generation sequencing (NGS) analyses, further validation of these need to be carried out in larger patient cohorts and variants characterized by other technical approaches. In summary, we demonstrate that circulating mRNA variants can potentially offer a viable liquid biopsy platform which on its own or in combination with cfDNA or protein biomarkers can significantly aid in surveillance, early detection and patient management.

## Data availability statement

All processed data in our study has been submitted in the manuscript in the form of figures and supplementary tables. Raw unprocessed FASTQ files can be accessed via a publicly available NCBI SRA database with accession number PRJNA907745 using this URL link: https://www.ncbi.nlm.nih.gov/bioproject/PRJNA907745.

## Ethics statement

The studies involving human participants were reviewed and approved by 1. Human Studies Subcommittee, Deptt. of veteran affairs Med Center, Univ. of Pennsylvania, PA. 2. IRB Capital Health cancer Center, NJ. The patients/participants provided their written informed consent to participate in this study.

## Author contributions

AS and TB conceptualized and designed the study. AS, TB and DZ developed methodology, planned and carried out experiments, analyzed data and provided interpretive insights. SZ helped carry out extracellular vesicle isolation. JL carried out mapping, GATK and SNPeff and other RNAseq analyses. TZ played a role in biostatistical analyses. DK and CD provided the clinical specimens and reviewed the data. AS and JL created the figures and AS drafted the manuscript. All authors contributed to the article and approved the submitted version.

## Supplementary material

Supplementary Figure 1 The KEGG pathway enrichment analysis conducted with R package cluster Profiler using “Over-representation analysis” based on hypergeometric distribution in the subset of genes with tumor-centric mutations. Over-representation of 578 genes with tumor-centric mutations in KEGG pathway/Gene Ontology (GO) terms.

Supplementary Table 1 Annotation and characteristics of top 1500 concordant high-risk variants found in tumors and plasma of HCC patients corresponding to Figure 3. Gene and genomic location, dbSNP rs numbers if available, variant effects and proportion/frequency in various clinical subsets are shown. The order of variants in the table corresponds to the order in the clustering heat map in Figure 3.

Supplementary Table 2 Annotation and characteristics of 1,369 high-risk tumor specific mRNA variants within the EVs of HCC patients corresponding to Figure 5C showing clustering dendrogram of samples. Gene and genomic location, dbSNP rs numbers if available, variant effects and proportion/frequency in various clinical subsets are shown. The order of variants in the table corresponds to the order in the clustering heat map in Figure 5C.

Supplementary Table 3 Annotation of 716 concordant variants identified simultaneously in two to three matching specimens (plasma, tumor, and/or vesicle) from same cancer patients, but not present in any of liver cirrhosis or healthy controls. Gene and genomic location, dbSNP rs numbers if available, variant effects and proportion/frequency in various clinical subsets are shown. The first 50 genes carrying the most of such high-risk concordant variants are shown in Fig 6B.

Supplementary Table 4 Annotation and characteristics of top 336 concordant high-risk variants found in tumors and plasma of CCA patients corresponding to Figure 7B. Gene and genomic location, dbSNP rs numbers if available, variant effects and proportion/frequency in various clinical subsets are shown. The order of variants in the table corresponds to the order in the clustering heat map in Figure 7B.

Supplementary Table 5 The frequency of tumor-related 5,155 variants in tumor/control samples were compared with Fisher's exact probability test under various group settings, i.e., HCC plasma vs. all the controls (normal plasma and cirrhosis plasma), HCC tumor vs all the controls (normal plasma and cirrhosis plasma), HCC vesicle vs all the controls (normal plasma and cirrhosis plasma), and all the HCC samples (plasma, tumor and vesicles) vs. all the controls (normal plasma and cirrhosis plasma).Variants are sorted on the basis of decreasing proportion in HCC tumors. Columns showing proportion of variants in HCC tumors (column DG) and p value between HCC tumors and all non tumor controls (column DQ) are highlighted in gray color. HCC - all includes all samples, tumors, plasma and extracellular vesicles from HCC patients. Tumor-all includes both HCC and CCA tumors. Control-all includes all samples corresponding tonon-cancer liver cirrhosis patients and those of normal healthy controls.

Supplementary Table 6 All the variants called from the 46 samples were filtered by depth (>=5) and quality score (>=20), and then annotated with SnpSift 5.1d and dbNSFP 4.3a to get clinvar information. There are 6,109 variants which had been reported in clinvar database. Clinical trait and clinical significance of each variant are indicated in the table. POS: location of variant;ID:dbSNP id; REF: Reference sequence; ALT: Altered sequence; QUAL: Quality of variant calling in phred score (-log10[probability of false calling]);INFO: information of variant including annotation by SnpSift.

Supplementary Table 7 Characteristics of HCC and CCA patients whose specimens were used. CH, NJ: Capital Health Cancer Center, NJ. UPEN, PA: Veterans hospital University of Pennysylvania, PA. Biochemed.
