# Supplementary figures and images for: Circulating messenger RNA variants as a potential biomarker for surveillance of hepatocellular carcinoma
Source: Front Oncol. 2022 Dec 13;12:963641. doi: 10.3389/fonc.2022.963641 (PMC9793749; doi:10.3389/fonc.2022.963641)

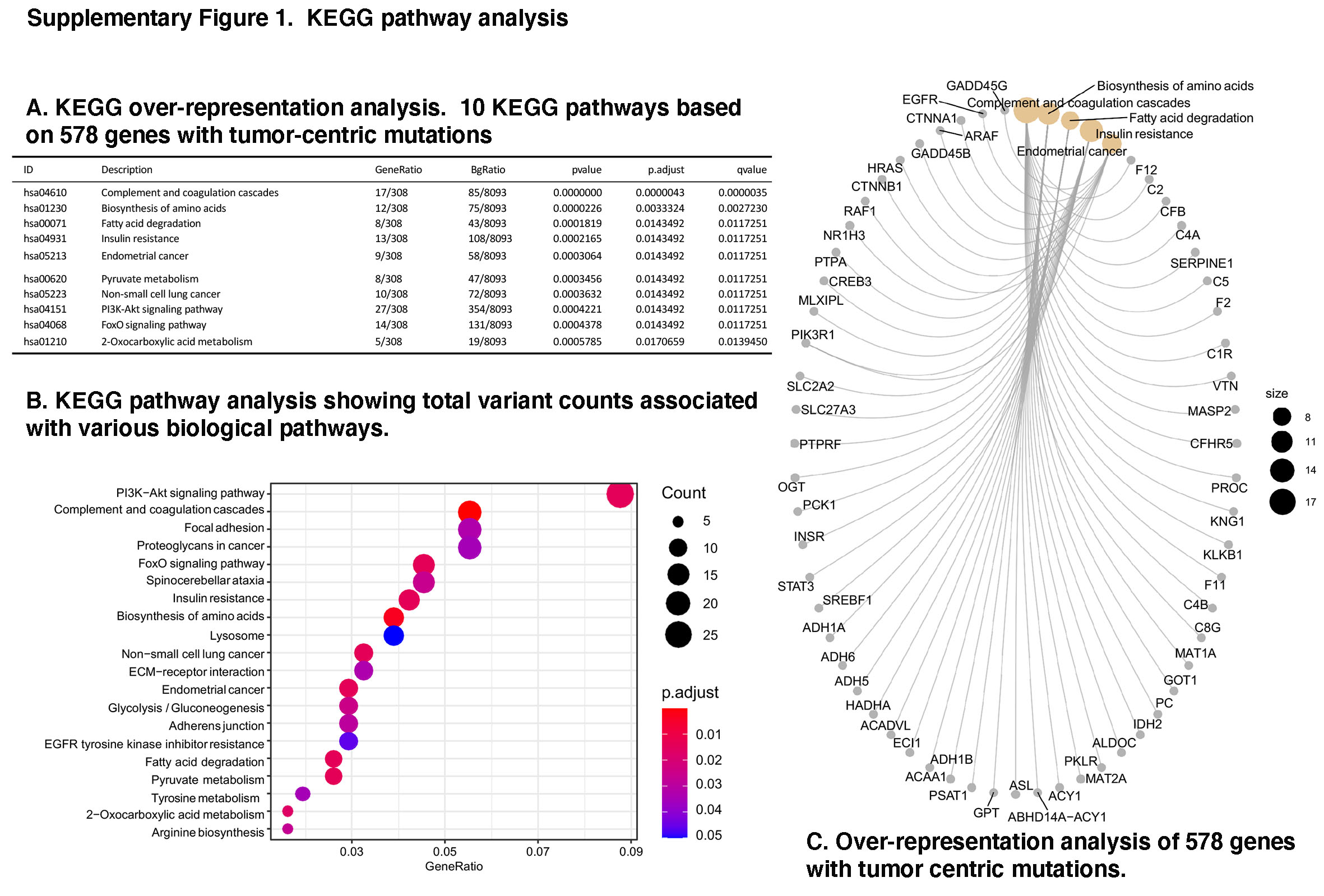

Supplement: Supplementary Figure 1 — The KEGG pathway enrichment analysis conducted with R package cluster Profiler using “Over-representation analysis” based on hypergeometric distribution in the subset of genes with tumor-centric mutations. Over-representation of 578 genes with tumor-centric mutations in KEGG pathway/Gene Ontology (GO) terms. [file Image_1.jpeg]
